# Clinical use of renal point-of-care ultrasound after extracorporeal shock wave lithotripsy

**DOI:** 10.1186/s13089-019-0141-8

**Published:** 2019-09-30

**Authors:** Luís Magalhães, Ramon Nogué

**Affiliations:** 1 Hospital da Luz – Arrábida, Praceta de Henrique Moreira 150, 4400-346 Vila Nova de Gaia, Portugal; 2 Universitat de Lleida, Plaça de Víctor Siurana, 1, 25003 Lleida, Spain

**Keywords:** Ultrasonography, Emergency medicine, Point-of-care testing, Diagnostic imaging, Extracorporeal shockwave therapy, Lithotripsy

## Abstract

**Background:**

Extracorporeal shock wave lithotripsy is widely used to treat symptomatic nephrolithiasis. Complications of this procedure can occur and point-of-care ultrasound can help to diagnose and manage some of these cases.

**Case presentation:**

A 61-year-old man was admitted to the hospital with intense right lumbar pain 24 h after being submitted to a extracorporeal shock wave lithotripsy. Bedside ultrasound showed a hyperechoic subcapsular lesion along the right kidney. This finding, along with the clinical examination, suggested the diagnosis of subcapsular renal hematoma. The patient was managed conservatively with clinical and ultrasound reassessments.

**Conclusions:**

This case shows the use of bedside ultrasound to diagnose a subcapsular renal hematoma as a complication of extracorporeal shock wave lithotripsy. However, the sensitivity is low and other image methods can be necessary to make the diagnosis.

## Background

Extracorporeal shock wave lithotripsy (ESWL) is the treatment of choice in many patients with symptomatic nephrolithiasis, mainly because of its effectiveness and lower invasiveness comparing to surgery [1]. However, complications of this procedure occur, namely incomplete stone fragmentation, renal parenchymal injury, a decline in glomerular filtration rate (rarely renal failure) and elevation in blood pressure [2, 3].

Point-of-care ultrasound (POCUS) is increasingly used by clinicians as a complement to the clinical evaluation, as a fifth pillar of the physical exam. Bedside evaluation of the abdominal cavity is well established, for example using the FAST or similar protocols.

We present a case where POCUS helped to diagnose and manage a patient with acute lumbar pain after ESWL.

## Description

A 61-year-old man was admitted to the hospital complaining of intense right lumbar pain with less than an hour of evolution. He had a history of hypertension, medicated and controlled, symptomatic nephrolithiasis and anxiety, without other significant medical history. He had been discharged from our hospital the day before following an ESWL for the treatment of 7-mm right renal stone. The procedure underwent without complications, approximately 2500 shocks, with apparent fragmentation of the stone. In our observation, the patient was afebrile, his blood pressure was 98/50 mmHg, heart rate 110 bpm and peripheral oxygen saturation 98%. On physical exam, he had pain on palpation of the right flank of the abdomen and a positive right Murphy sign. Physical examination was otherwise unremarkable. Blood tests at admission were: hemoglobulin 11.5 g/dL (N 13.5–17.5 g/dL), total white blood cell 10.010/μL (N 3.500–11.000/μL), 77.8% neutrophils (N 45–75%), platelets 210.000/μL (N 125.000–400.000/μL), International Normalized Ratio 1.11, creatinine 1.35 mg/dL (N 0.6–1.3 mg/dL), urea 38 mg/dL (N 22–52 mg/dL) and C-reactive protein 6.4 mg/dL (N < 5 mg/dL).

**Fig. 1:**
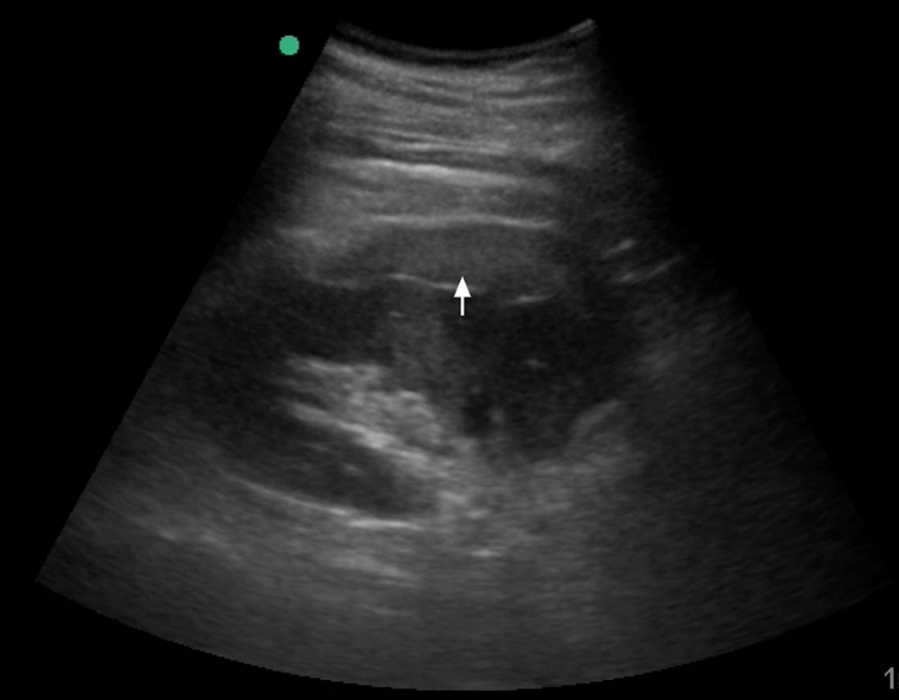
Point-of-care Ultrasound of the right kidney. Sixty-one-year-old male 24 h after ESLW presenting with acute right lumbar pain. Point-of-care ultrasound: longitudinal view of the right kidney using a 5-MHz curvilinear probe showing a subcapsular hyperechogenic collection of fluid, corresponding to an acute subcapsular renal hematoma (arrow)

**Fig. 2:**
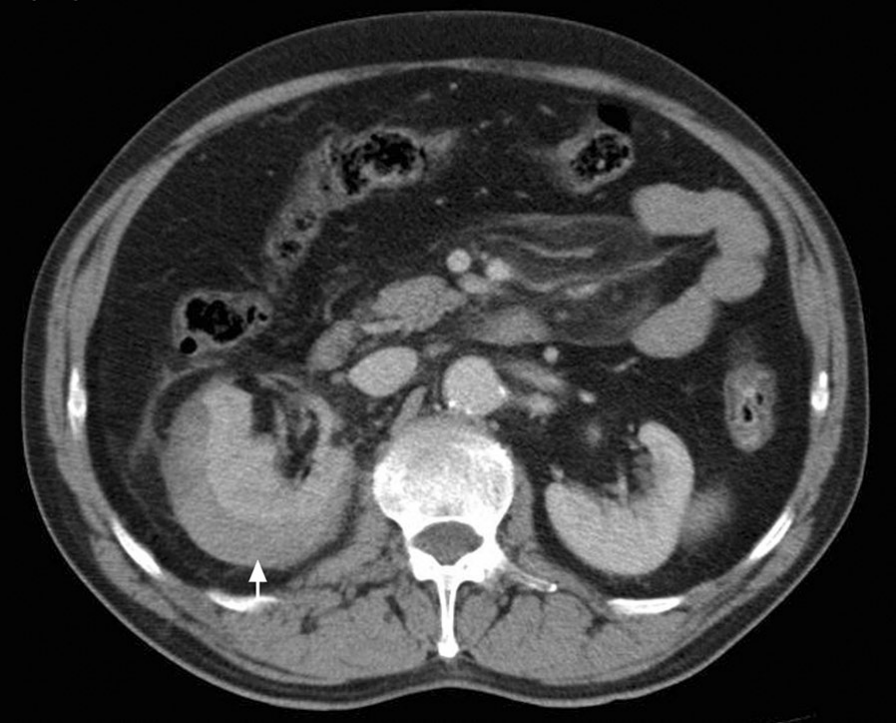
CT scan of the abdomen. Sixty-one-year-old male 24 h after ESLW presenting with acute right lumbar pain. Contrast CT showing a subcapsular collection in the right kidney (arrow), compatible with subcapsular renal hematoma, without other renal or abdominal complications

A bedside ultrasound was performed which showed a hyperechoic subcapsular collection along the right kidney (Fig. 1). The remaining exam was unremarkable, including no evidence of intraperitoneal fluid and absence of nephrolithiasis or pyelocalyceal dilatation. The diagnosis of subcapsular renal hematoma secondary to ESWL was made. A Computerized Tomography (CT) scan to exclude other complications showed the presence of subcapsular lesion suggestive of subcapsular renal hematoma (Fig. 2), without other complications.

Conservative measures were adopted, with a special focus on pain management. The patient was re-evaluated using POCUS to exclude any additional complication and was discharged 24 h after.

## Discussion

ESWL is increasingly used in clinical practice, leading to a greater number of potential complications. The parenchymal injury is originated by the shock waves that affect the blood vessels, leading to hematomas. The degree of damage depends on the technique, kidney morphology, patient co-morbidities and stone characteristics [4–6]. Most of them are asymptomatic, but a few present with ipsilateral pain, as described above. Clinical evaluation, including physical evaluation, is important but frequently not enough to clearly differentiate between the most frequent complications of ESLW. It is our understanding that the addition of POCUS to the clinical evaluation can improve diagnostic accuracy of the possible complications, as this case shows. Ultrasound has many advantages, including a favourable safety profile, without ionizing radiation, increasing availability and can be easily performed multiple times on the bedside.

In this particular scenario, POCUS helped in the differential diagnosis of lumbar pain after ESWL, favoring subcapsular renal hematoma against other potential diagnoses, including incomplete stone fragmentation and, less frequently, kidney rupture [7].

The sonographic appearance of a subcapsular renal hematoma is usually a crescent-shaped or ellipsoid hyper-, iso- or hypoechoic lesion located around the kidney in the subcapsular space. Hematomas can appear heterogenous and hyperechogenic in the acute phase, becoming more hypoechoic or cystic over time [8, 9]. Technically, the kidneys are usually well identified using a standard curvilinear probe. The visualization of the hyperechoic renal capsule helps to differentiate between the fluid in the intraabdominal cavity and the hematoma, as subcapsular hematomas are between this capsule and the cortex. Subcapsular renal hematomas can be more difficult to see in the acute phase because they may have the same echogenicity of the renal cortex.

It is important to note that the sensitivity of renal ultrasound for subcapsular renal hematomas is low compared to CT [10]. So, a normal POCUS should not be used to rule out a subcapsular renal hematoma (or other complications). When there is a clinical suspicion, a more sensitive exam like a CT scan should be obtained.

Treatment of these conditions depends on hemodynamic stability. Most of the patients, like this one, stay hemodynamically stable and the treatment should be conservative, consisting of clinical observation and exclusion of other complications [11]. POCUS can also be a useful tool in this setting, as it allows for continuous monitoring, especially in case of worsening of the symptoms. More severe cases may require blood transfusions, percutaneous drainage [12], that can be guided using ultrasound, or even, in very rare cases, nephrectomy.

## Conclusions

ESLW is increasingly used to treat nephrolithiasis. Even though it is considered a safe method, it is associated with an important number of complications. POCUS, in addition to a complete clinical evaluation, can help to differentiate some of the most frequent complications quickly and safely, including subcapsular renal hematoma, as demonstrated by this case. However, ultrasound has a low sensitivity for this type of hematoma and should not substitute other image methods when needed.

## Data Availability

Data sharing is not applicable to this article as no datasets were generated or analyzed during the current study.

## Abbreviations

CT: Computerized Tomography
ESLW: extracorporeal shock wave lithotripsy
POCUS: point-of-care ultrasound
